# Orofacial function and temporomandibular disorders in Parkinson’s Disease: a case-controlled study

**DOI:** 10.1186/s12903-023-03051-6

**Published:** 2023-06-12

**Authors:** Sara Baram, Carsten Eckhart Thomsen, Esben Boeskov Øzhayat, Merete Karlsborg, Merete Bakke

**Affiliations:** 1 Department of Odontology, Faculty of Health and Medical Sciences, University of Copenhagen, 20 Nørre Allé, Copenhagen N, DK-2200 Denmark; 2 Department of Neurology, Bispebjerg University Hospital, Copenhagen, Denmark

**Keywords:** Parkinson’s disease, Orofacial function, DC/TMD, Mastication, Swallowing, Electromyography, Xerostomia, Drooling, NOT-S, Neurological diseases

## Abstract

**Background:**

The difficulties and challenges faced by people with Parkinson’s disease (PD) in performing daily orofacial function are not systematically investigated. In this study, specific orofacial non-motor and motor symptoms and functions were systematically examined in PD patients in comparison to a matched control group.

**Methods:**

The clinical case-controlled study was conducted from May 2021 to October 2022 and included persons with PD and age- and gender-matched persons without PD. The participants with PD were outpatients diagnosed with PD at the Department of Neurology at Bispebjerg University Hospital in Copenhagen, Denmark. The participants underwent a systematic clinical and relevant self-assessment of the orofacial function and temporomandibular disorders (TMD). The primary outcomes were objective and subjective assessments of the general orofacial function, mastication, swallowing, xerostomia and drooling. The secondary outcomes were the prevalence of TMD and orofacial pain. The difference in outcome measures between the two groups was analysed using chi-square and Mann–Whitney U test.

**Results:**

The study included 20 persons with PD and 20 age- and gender-matched persons without PD. Both objectively and subjectively, persons with PD had poorer orofacial function than the control group. Persons with PD had also a significantly more severe limitation of jaw mobility and jaw function. The objective masticatory function was also significantly reduced for persons with PD compared to the control group, and 60% of persons with PD found it difficult to eat foods with certain consistencies while 0% of the control group reported that problem. Persons with PD could swallow less water per second and the average swallowing event was significantly longer for PD persons. Even though PD persons reported more xerostomia (58% for persons with PD and 20% for control persons), they also reported significantly more drooling than the control group. Additionally, orofacial pain was more prevalent in PD persons.

**Conclusions:**

Persons with PD have a compromised orofacial function. Furthermore, the study indicates a link between PD and orofacial pain. In order to screen and treat persons with PD accordingly, healthcare professionals should be aware of and address these limitations and symptoms.

**Trial registration:**

The trial was approved by the Regional Committee on Research Health Ethics of the Capital Region (H-20,047,464), the Danish Data Protection Agency (514 − 0510/20-3000), and registered at ClinicalTrials.gov (NCT05356845).

## Background

Parkinson’s disease (PD) is a chronic, age-related neurodegenerative disease. PD is caused by the slow loss of brain cells in the substantia nigra, particularly in cells that release dopamine. The deficiency of dopamine and other neurotransmitters results in motor symptoms (slow movement, tremors, stiffness, and unbalanced walking) and a wide range of non-motor symptoms (cognitive impairment, mental health disorders, pain, and other sensory disturbances) [1]. The progression of these symptoms and complications significantly lowers daily functioning and quality of life, leading to high rates of disability, care needs, and increased demand on families, caregivers, and society [2, 3].

Both the motor and non-motor symptoms have an impact on the dental and oral health of persons with PD. Several studies have observed fewer teeth, more caries, increased periodontal disease, and poorer oral hygiene in persons with PD compared to age-matched controls [4–9]. PD persons also often suffer from xerostomia, dysphagia, and drooling, and besides the direct impact of the PD symptoms, impaired oral health is also due to the medical management of PD [10, 11]. The decline in oral status is correlated with the progression of the disease [9]. PD and other neurological conditions can also affect the facial and masticatory muscles, which can reduce jaw mobility and result in problems with orofacial function as well as temporomandibular disorders (TMD) [6, 9, 12, 13]. TMD is the second most occurring musculoskeletal disorder that causes pain and disability [14–16]. To facilitate research and clinical practice with respect to TMD, reliable tools to assess disease state are developed for clinical and research use under the heading Diagnostic Criteria for Temporomandibular Disorders (DC/TMD). This includes more general instruments to identify jaw-related functional limitations which may be useful in PD [14]. However, this aspect of PD has largely been described by non-clinical observational studies or questionnaires. As a result, there is a lack of knowledge about the challenges and problems of persons with PD in their daily orofacial function.

No studies have conducted *both* a relevant self-assessment survey, a systematic clinical examination of the orofacial function *as well as* recordings of TMD in persons with PD. It is important to establish valid data which can enlighten these aspects adequately and provide insight and information to health and dental care professionals on the subject, so they can plan their assistance appropriately. Due to the complexity of the disease and the patients’ heterogeneous and individual symptoms, persons with PD are often offered multidisciplinary management of their disease. Besides medical management, this includes occupational therapy, physiotherapy, voice training etc. New knowledge concerning problems with orofacial function can provide ideas on how the improvement of this aspect also can be implemented in this multidisciplinary approach and may be a contributing factor to a better quality of life.

This study aimed to investigate specific orofacial non-motor and motor symptoms and functions in persons with PD compared to a matched control group. This will be conducted in the form of combined objective clinical examinations and self-assessments of orofacial function and TMD.

## Methods

### Study design, timeline, setting and participants

This clinical, case-controlled study was conducted from May 2021 to October 2022 as a collaboration between the Department of Odontology, University of Copenhagen, Denmark, and the Department of Neurology, Bispebjerg University Hospital, Copenhagen, Denmark. All PD participants were outpatients at the Department of Neurology and diagnosed with PD by a neurologist prior to recruitment. They were informed about the study, recruited, and neurologically examined by a senior consultant in neurology, author MK. Subsequently, a dental and orofacial examination consisting of observations, questionnaires, and clinical and electromyographic examinations was performed by a dentist with special training in clinical oral physiology, author SB. An age- and gender-matched control group was recruited among spouses of the PD participants and by flyers at the hospital, University of Copenhagen, and public libraries.

An earlier study [6] suggests that PD persons have a 50% risk of suffering from orofacial symptoms when examined by the Nordic Orofacial Test – Screening (NOT-S) relative to the control group which is assumed to have a 10% risk of suffering from orofacial symptoms. With a power of 80% and a significance level of 0.05, we needed 19 participants in each group. We, therefore, included 20 participants with PD and 20 participants in the control group.

The PD participants were appropriately medicated, and their disease was well-managed by the neurologist. The inclusion criteria for both PD participants and controls were to understand the participant information and fully cooperate during the examinations. Furthermore, they had to transport themselves to and from the Department of Odontology and sit upright in a dental chair during the examination.

The exclusion criteria for all participants were: cognitively affected persons unable to understand the information given and not able to cooperate during the study, persons diagnosed with Sjögren’s syndrome, persons with implanted electronic devices (pacemaker, DBS, and the like), and/or persons who receive or have received radiation in the head/neck region as part of cancer treatment.

### Characteristics and classification of the participants

A clinical dental examination was performed and included registration of (1) the number of teeth including fixed prosthetic restorations e.g., crowns, bridges, and implants, (2) posterior occlusal contacts according to the Eichner Index (classified in A: having occlusal contact in all four supporting zones, B: having occlusal contact in one to three supporting zones, and C: having no occlusal contact at all) which is also associated with occlusal force and masticatory performance [17], and (3) removable dentures. For persons with PD, the neurological assessment included the duration of PD in years and the progression of PD according to the modified Hoehn and Yahr scale (H&Y) [18].

### Outcomes

#### Assessment of orofacial function, orofacial pain, and TMD

A systematic assessment of TMD was performed according to the Diagnostic Criteria for TMD (DC/TMD) [14] including the Symptom Questionnaire (SQ) and full clinical examination from Axis (I) The participants were also asked to fill out the Jaw Functional Limitation Scale (JFLS-8) and Chronic Pain Grade Scale (GCPS) from Axis (II) The JFLS-8 was used to assess the functional status of the masticatory system and the functional limitations of the jaw and produced a score between 0 and 80 with a high value suggesting a high degree of limitation of the jaw function. The GCPS was used to assess chronic facial pain conditions, by measuring the overall severity of chronic facial pain, as well as pain intensity and pain-related disability. The pain severity was graded into 4 hierarchical classes: Grade I, low disability-low intensity; Grade II, low disability-high intensity; Grade III, high disability-moderately limiting; and Grade IV, high disability-severely limiting [19]. Grade I, II, III IV was merged and categorized as having chronic facial pain. Furthermore, the participants were categorised as having non-specific orofacial pain and/or headache if they answered yes to (1) SQ number 3 and Examination Question 1a (from the DC/TMD), and/or (2) SQ-5 and Examination Question 1b (from the DC/TMD) but did not fit the rest of the diagnostic criteria for DC/TMD. Also, the participants were asked to answer questions regarding physical pain from the Oral Health Impact Profile-14 (OHIP-14) [20]: *Have you had painful aching in your mouth?* and - *Have you found it uncomfortable to eat any foods because of problems with your teeth, mouth, or dentures?*

A screening for orofacial dysfunction was performed according to the Nordic Orofacial Test – Screening (NOT-S). This resulted in a score between 0 and 12 indicating the degree of orofacial dysfunction [21]. Participants were also asked to assess their masticatory function subjectively by answering a question from NOT-S: *Do you find it difficult to eat foods with certain consistencies?* and two questions regarding physical ability from the OHIP-14: *Has your diet been unsatisfactory because of problems with your teeth, mouth, or dentures?* and *Did you interrupt meals due to problems with your teeth, mouth, or dentures?* The masticatory efficiency was assessed by the amount of time, counted in seconds/s, it took the participants to chew and swallow a standardized piece of apple (10 g) [6]. The guiding reference interval is 7–53 s [22]. The swallowing capacity was assessed by the “Timed water swallow test”, which yielded how many ml participants can swallow per second [23]. Furthermore, an electromyographic (EMG) examination with surface electrodes during chewing and swallowing was performed bilaterally of the anterior temporal muscles, the masseter muscles, and the anterior belly of the digastric muscles [24]. The duration of the chewing cycle (in milliseconds) was measured by EMG during the chewing of apple slices and was determined as the average of 5 strokes in 3 chewing sequences. The right anterior temporal muscle was used as the reference muscle, and the definition of a chewing cycle was from the start of temporal activity in one closing phase to the start of temporal activity in the next closing phase. The guiding reference interval is 441–785 ms [22]. The swallowing duration (in milliseconds) was assessed by swallowing 2 ml of water during EMG recording and using the right anterior temporal muscle as a reference muscle. The start of a swallow was defined as the point at which the EMG trace exceeded the threshold level and the ending of a swallow was defined as the point at which a trace returned below the threshold level. Furthermore, it was noticed if the participant had more than one swallowing in the same event (double or triple swallowing of 2 ml of water). All the EMG data were collected and analysed (after amplification and filtering), using an 8-channel EMG system (gain 500 − 10,000; high-pass filter at 20–50 Hz and low pass at 1 kHz). The amplified and filtered EMG were digitized with 12-bit resolution and 2.5 kHz sample rate. Reusable, bipolar surface tin electrodes with electrode paste measuring 10.0 × 3.0 × 1.5 mm for the anterior temporal and masseter muscles and 5.0 × 3.0 × 1.5 mm for the anterior belly of the digastric muscles were used to record the muscle activity. The electrodes were positioned perpendicular to the muscle fibre direction after cleansing the skin with alcohol to lower the impedance.

Finally, a subjective assessment of xerostomia and drooling was performed, as xerostomia may influence masticatory activity, and swallowing problems may provoke drooling. Symptoms of dry mouth were assessed by using a questionnaire with four levels of severity; 0) no feeling of dry mouth, (1) slight feeling of dry mouth, (2) severe feeling of dry mouth, and (3) an annoying feeling of dry mouth that makes speech difficult [25], score 1,2 and 3 were merged and categorised as “Feeling of dry mouth”. Subjective assessment of drooling was performed by a questionnaire, where the participants were asked to rate their drooling in terms of frequency and quantity [26]. The total score is between 2 and 9, where 2 corresponds to no problems with drooling and 9 corresponds to severe drooling. Scores greater than 3 were categorised as having drooling problems.

### Statistics

Data were analysed using IBM SPSS Statistics 27. Initially, a descriptive analysis of the characteristics of the study population was performed and included age, gender, number of teeth and posterior occlusal contacts. The outcome measures were divided into general orofacial function, mastication, swallowing and xerostomia and drooling. The difference between the two groups regarding characteristics and outcome measures was analysed using Mann–Whitney U test for continuous and ordinal variables and chi-square for categorical variables, as the data were not normally distributed. Also, orofacial pain and TMD diagnosis were described but not statistically compared between the study and control population as the participants can have multiple DC/TMD diagnoses. The participants with PD were ultimately stratified according to the duration of their PD into three categories. The first category included 6 persons who had PD for 3–6 years, the second category included 7 persons who had PD for 7–11 years and the last category included 7 persons who had PD for 12 years or more.

## Results

### Characteristics

The study group included 40 persons, 20 with PD and 20 without PD. The median age for the PD group was 68.5 years and for the control group 67.0 years. The gender distribution was the same for both groups with 6 males and 14 females. No significant differences were found between the PD and control groups concerning age, gender, number of teeth including fixed prosthetic restorations, and posterior occlusal contacts (Table 1). None of the participants had posterior removable dentures, but one participant in the control group had a partial denture only involving the front teeth. For the PD group, the years with PD ranged from 3 to 23 years and the progression according to H&Y was from 1 to 4 (Table 1).

**Table 1:** Characteristics of the study population

	PD population	Control population
**Study population**	20	20
**Age in years**		
Median (range)	68.5 (35–80)	67.0 (35–83)
**Gender**		
Male	6	6
Female	14	14
**Number of teeth** Median (range)	27 18–31	27 22–31
Eichner index*		
A B C	85% 15% 0%	95% 5% 0%
**Years with PD**		
Median (range)	10 (3–23)	-
**H&Y** ^**+**^		
Median (range)	3 (1–4)	-

*Eichner: a classification system based on the presence of occlusal contacts in the premolar and molar regions

^+^H&Y: Modified Hoehn and Yahr scale

### Orofacial function (Table 2)

**Table 2:** Orofacial function in persons with PD and control persons

	PD population	Control population	p-value
**General orofacial function**			
NOT-S sum*			
**Median (range)**	3 (0–7)	0 (0–2)	**p < .001** ^**a**^
**Maximum jaw opening, unassisted in mm**			
Median (range)	50 (37–60)	54 (41–60)	p = .149^a^
**Laterotrusion, mean in mm to each side**			
Median (range)	9.25 (3.5–13.5)	11.5 (10–14)	**p = .006** ^**a**^
**Protrusion in mm**			
Median (range)	7.5 (1–11)	9 (5–13)	p = .134^a^
**JFLS sum** ^+^			
Median (range)	4.5 (0–33)	0 (0–4)	**p < .001** ^**a**^
**Mastication**			
**Do you find it difficult to eat foods with certain consistencies?**			
No	40%	100%	
Yes	60%	0%	**p < .001** ^**b**^
**Question 7 (OHIP-14**^α^): **Has your diet been unsatisfactory because of problems with your teeth, mouth or dentures?**			
No	42%	95%	
Yes (score 1–4)	58%	5%	**p = .006** ^**b**^
**Question 8 (OHIP-14**^α^): **Have you had to interrupt meals because of problems with your teeth, mouth, or dentures?**			
No	79%	100%	
Yes (score 1–4)	21%	0%	**p = .030** ^**b**^
**Masticatory efficiency of 10 g crisp apple chewing in seconds**			
Median (range)	25 (14–80)	19 (10–31)	**p = .040** ^**a**^
**EMG° crisp apple chewing cycle in ms**			
Median (range)	580 (434–920)	623 (429–934)	p = .529^a^
**Swallowing**			
**Swallowing capacity, mL water pr. second**			
Median (range)	11 (2–25)	19 (9–50)	**p < .001** ^**a**^
**EMG° 2 mL water swallowing time in ms**			
Median (range)	1847 (1233–2867)	1433 (800–1700)	**p < .001** ^**a**^
**EMG° swallowing pattern of 2 mL water**			
Normal swallowing	50%	100%	
Double, triple or distorted swallowing	50%	0%	**p < .001** ^**b**^
**Salivary flow and drooling**			
**Xerostomia**			
No feeling of dry mouth	42%	80%	
The feeling of dry mouth	58%	20%	**p = .015** ^**b**^
**Drooling, subjective (2–9)**			
Median (range)	4 (2–5)	2 (2–2)	**p < .001** ^**a**^

Significance level: **p < .05**

a: Mann-Whitney U Test

b: χ2-test

*NOT-S: Nordic Orofacial Test – Screening, score 0–12

^+^JFLS: Jaw Functional Limitation Scale − 8, score 0–80

^α^OHIP-14: The Oral Health Impact Profile − 14

° EMG: Electromyography

#### General orofacial function

**Fig. 1:**
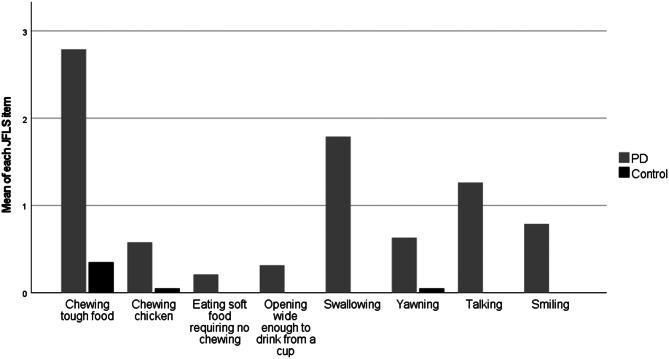
Mean score of the 8 items of the Jaw Functional Limitation Scale (JFLS-8) from the DC/TMD in persons with PD and the control group

**Fig. 2:**
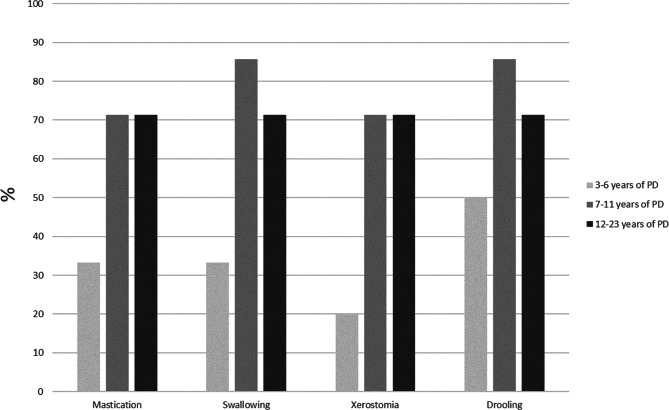
Self-reported problems with the orofacial function among the participants with PD. The participants were divided into three groups according to the duration of their PD.

The NOT-S score was significantly higher regarding the scores in the interview, the examination and the total sum. Thus, the PD persons had a median total sum of 3, while non-PD persons had a median total sum of 0. The jaw mobility (maximum unassisted opening, laterotrusion and protrusion) was generally less for the PD group, but only the laterotrusion was statistically lower and significant. Self-assessment of the functional limitations of the jaw (JFLS) was significantly more severe for the PD persons with a median score of 4.5 compared to a median score of 0 for the control persons. Figure 1 depicts the 8 items of the JFLS Scale and shows that the most severe limitation is chewing tough food, swallowing and talking. In addition Fig. 2 illustrates that the self-reported problems with the orofacial function in terms of mastication, swallowing, xerostomia, and drooling seem to worsen over time.

#### Mastication

The subjective assessment of mastication was significantly worse for the PD persons compared to the control group as 60% found it difficult to eat foods with certain consistencies, 58% felt their diet has been unsatisfactory and 21% had to interrupt meals. The objective masticatory function was also significantly reduced for persons with PD compared to the control group, as it took an average of 8 s longer to chew a standardized apple slice compared to the control group, and for two of the PD persons, the chewing time was above one minute and thereby above the guiding reference interval. However, EMG recordings showed no significant difference in the length of the chewing cycle between the two groups, and three participants in each group had cycles above the guiding reference interval.

#### Swallowing

Control persons were able to swallow approximately 10 mL more water per second than persons with PD. EMG recordings showed that the average swallowing event was significantly longer for PD persons. Furthermore, 50% of persons with PD had double, triple or distorted swallowing activity during the swallowing of 2 ml water, instead of a well-defined single event as recorded from the participants in the control group.

#### Xerostomia and drooling

Subjectively, 58% of people with PD felt that they have dry mouths compared to only 20% in the control group. Even though PD persons reported more xerostomia, they also reported more drooling and scored an average of 2 points more on the drooling scale.

### Orofacial pain and DC/TMD assessment

Diagnosis according to the DC/TMD is displayed in Table 3 and shows no marked difference between the study and control population. The same applies to non-specific non-TMD-related orofacial pain and/or headache. Two persons with PD were diagnosed with myogenic TMD but none were in the control group. Disc displacement diagnoses were relevant in four persons with PD and in one control person, and concerning degenerative joint diseases, this diagnosis was given in three persons with PD and five control persons. It should be noted that a participant can have more than one DC/TMD diagnosis. In terms of GCPS and physical pain according to OHIP-14, 25% of persons with PD experienced chronic facial pain, 42% have had painful aching in the mouth in the last 3 months, and 56% have found it uncomfortable to eat any foods in the last 3 months. The GCPS and physical pain domain from the OHIP-14 was all more pronounced for persons with PD, but only question 4 from OHIP-14, regarding being uncomfortable eating foods, was significant.

**Table 3:** DC/TMD and orofacial pain in the study population. Participants can have one or more DC/TMD diagnoses and/or non-specific orofacial pain and/or headache

	PD population	Control population
DC/TMD diagnosis or non-specific orofacial pain and/or headache*		
No DC/TMD diagnosis or non-specific orofacial pain and/or headache	9	11
**Degenerative joint disease uni- or bilaterally** ^+^	3	5
**Myalgia masseter and/or temporalis** ^+^	2	0
**Disc displacement with reduction uni- or bilaterally** ^+^	4	1
**Arthralgia uni- or bilaterally** ^+^	1	0
Non-specific orofacial pain and/or headache*	5	3
**Chronic Pain Grade Scale (GCPS)**		
No chronic pain Chronic pain, Grade I, II, III, VI l	75% 25%	95% 5%
**Oral Health Impact Profile (OHIP-14 physical pain domain)**		
Question 3: Have you had painful aching in your mouth?		
No Yes (score 1–4)	58% 42%	70% 30%
Question 4: Have you found it uncomfortable to eat any foods because of problems with your teeth, mouth, or dentures?		
No Yes (score 1–4)	44% 56%	85% 15%

* Non-specific orofacial pain and/or headache: If the participants answered yes to (1) Symptom Questionnaire 3 and Examination Question 1a, and/or (2) Symptom Questionnaire 5 and Examination Question 1b, but did not fit the rest of the diagnostic criteria for DC/TMD.

^+^Diagnosed according to DC/TMD

## Discussion

People with PD have poorer orofacial function than the control group, both clinically and subjectively, even though they were matched on age, gender and teeth status. The poorer orofacial function was present in several individual functions such as jaw function, mastication and swallowing.

The foremost affected orofacial function, both objectively and subjectively, was swallowing. Difficulty in swallowing, that is dysphagia, can partly be explained by restriction and rigidity of the orofacial and oropharyngeal muscles and also by xerostomia. The condition can become serious and sometimes lead to malnutrition, dehydration, and aspiration [27]. The masticatory function was just significantly worse for people with PD. However, when examining the range for how long it took to chew a standardised slice of apple, it took up to 1.5 min for some persons with PD, which shows a major inhibition of the masticatory function compared to control subjects. These findings fit well with other studies showing that objective and subjective orofacial function is reduced in persons with PD [6, 9, 28]. However, another study shows that both reduced masticatory function and jaw mobility can be partially prevented or improved with physiotherapeutic orofacial exercises [29, 30].

In terms of TMD diagnoses, there is no difference between people with and without PD. This fits well with other non-clinical epidemiological studies [31], which show that the prevalence of orofacial pain conditions in PD persons is similar to that in the general population. However, Korean and Taiwanese epidemiological studies show a higher risk for TMD in people with PD, but no standardized diagnostic instrument for TMD was used in these studies [32, 33]. Another clinical study demonstrated that the prevalence of TMD among persons with PD was approximately 20%, but the study was not controlled [34]. Even though we could not find a strong association between PD and TMD according to the DC/TMD, there was a difference in non-specific orofacial pain, GCPS and the physical pain domain from OHIP-14, which could indicate that even though they do not have more DC/TMD diagnoses, they suffer to a greater degree from non-specific facial pain. This has also been reported in several studies as chronic pain is one of the many non-motor symptoms of PD [35]. A Dutch study also showed an association between PD and non-specific orofacial pain and non-specific possible TMD pain [36].

The participants with PD in our study were well-medicated accordingly to their PD status, the medical treatment included: dopamine agonists, levodopa, monoamine oxidase B inhibitors, and anticholinergic agents, which all have several side effects that might affect oral health and orofacial function. Levodopa and dopaminergic treatment may cause new uncontrollable and involuntary movements of the face and limbs (dyskinesia) which again can affect mastication and swallowing, while anticholinergics can cause dry mouth [37]. It is assumed, that all medications for PD can cause psychiatric side effects such as confusion, hallucination, and memory problems [37], which can make it difficult to maintain sufficient daily oral hygiene and regular dental visits. It is difficult to assess how much of the oral health and orofacial function is expected to be reduced due to the disease itself or the medication, but doctors must be aware of these side effects when they prescribe the medication.

The inferior orofacial function of persons with PD can have clinical implications. Early PD patients frequently underreport non-motor complaints because they do not associate them with their neurological condition. Patients and medical professionals frequently disregard these symptoms in favour of concentrating on the motor signs that led to the patient’s visit with the neurologist [35]. However, the consequences of orofacial pain and TMD can be severe, as studies indicate that TMD is related to depression and has a detrimental impact on oral health-related quality of life [38, 39]. This is in addition to the fact that PD itself is linked to higher rates of depression and several other comorbidities [35]. The findings of this study underline the important relevance of screening and/or examination for orofacial function and pain in persons with PD. It is important for dental care professionals to have a status of jaw mobility and jaw limitation so that they can give correct advice regarding the maintenance of oral hygiene and exercise in due diligence, especially when it comes to dental prophylaxis and treatment. It is also important for other healthcare professionals to be aware of these limitations and symptoms, thereby treating the patient as a whole person. The clinical aspect of this study will familiarize the limitation in orofacial function and TMD symptoms for health care professionals. This will assist neurologists, dentists, and other clinicians to recognise this aspect of PD so prevention and treatment can be implemented in a multidisciplinary approach surrounding the patient.

### Strengths and limitations

The strength of this study is the systematic clinical, electromyographic and subjective examination of multiple aspects of the orofacial function and temporomandibular dysfunction. The limitation is the relatively low number of participants in the trial. Because of this, it has not been possible to adjust statistically for covariates. We included persons with PD with a wide range of disease severity and years with PD, but a limitation was that PD persons who participated in the study were also resourceful people who had the energy to participate and could easily transport themselves to the setting at the University. The severely affected persons with PD, both mentally and physically, declined the offer to participate in the study. Thus, there is a selection bias among the participants with PD, which probably underreported the problems, both subjectively and objectively.

## Conclusion

This study systematically demonstrates that persons with PD have a compromised orofacial function compared to a matched control group. Furthermore, the study suggests an association between PD and orofacial pain. Healthcare professionals should be aware of these facts and ensure that persons with PD are screened for orofacial dysfunction and pain and treated accordingly.

## Data Availability

The dataset used and/or analysed during the current study is available from the corresponding author upon reasonable request, but a transfer of data must comply with the General Data Protection Regulation (GDPR) of the European Union (EU).

## Abbreviations

PD: Parkinson’s disease
TMD: Temporomandibular disorders
DC/TMD: Diagnostic Criteria for Temporomandibular disorders
H&Y: Modified Hoehn and Yahr scale
SQ: Symptom Questionnaire
JFLS-8: Jaw Functional Limitation Scale − 8
GCPS: Chronic Pain Grade Scale
OHIP-14: Oral Health Impact Profile − 14
NOT-S: Nordic Orofacial Test – Screening
EMG: Electromyography
